# Diagnostic challenge in diagnosing bilateral breast metastases from mediastinal neuroendocrine tumor: A case report

**DOI:** 10.1016/j.amsu.2020.11.035

**Published:** 2020-11-11

**Authors:** Kheng Hooi Chan, Chang Haur Lee, Siti Zubaidah Sharif, Firdaus Hayati, Sugunah Sallapan

**Affiliations:** a Department of Surgery, Hospital University Kebangsaan Malaysia Medical Centre, Kuala Lumpur, Malaysia; b Department of Breast and Endocrine Surgery, Queen Elizabeth Hospital 2, Ministry of Health Malaysia, Kota Kinabalu, Sabah, Malaysia; c Department of Surgery, Faculty of Medicine and Health Sciences, Universiti Malaysia Sabah, Kota Kinabalu, Sabah, Malaysia; d Department of Pathology, Queen Elizabeth Hospital, Ministry of Health Malaysia, Kota Kinabalu, Sabah, Malaysia

**Keywords:** Breast neoplasms, Diagnostic imaging, Mediastinal neoplasms, Neuroendocrine tumours

## Abstract

**Background:**

Metastatic neuroendocrine tumours (NETs) to the breast are very rare entities.

**Case presentation:**

A 26-year-old lady presented with anterior neck swelling with symptoms of superior vena cava syndrome for 6 months. Imaging study revealed a mediastinal mass which was preceded with core biopsy which was consistent with high-grade small cell NETs. Despite second-line adjuvant chemotherapy and radiotherapy, her disease became advanced which was confirmed via restaging scan. There were bilateral breast lesions discovered during the scan which was deemed to be metastatic NETs histologically. Despite prompt initiation of treatment, she succumbed 1 year after the radiotherapy due to disease progression.

**Conclusion:**

High suspicion of an index is needed for diagnosis when patients with known primary NETs present with suspicious breast lesions. Triple assessment is mandatory, however histopathology assessment and immunohistochemistry staining are the mainstay of diagnosis.

## Highlights

• Metastatic breast neuroendocrine tumours are very rare entities especially when they arise from the mediastinum.
• High suspicion of an index is needed for diagnosis when patients with known primary neuroendocrine tumours presented with suspicious breast lesions.
• Any breast pathology requires a triple assessment including secondary breast neuroendocrine tumours.

## 1 Introduction

Neuroendocrine tumours (NETs) are an abnormal growth of the enterochromaffin cells. They are slow-growing tumours found mostly in the gastrointestinal tract and bronchopulmonary region [1]. NETs of the mediastinum are rare and usually metastasize to the lungs and bones [2]. Primary breast NETs are uncommon and primary NETs metastasizing to the breast are very rare [3]. They constitute only about 7% of all distant metastatic tumours to the breast [4]. The breast is an uncommon site for metastases from other non-mammary malignancies as the breast contains large areas of fibrous tissue with a relatively poor blood supply distribution. Patients with breast metastases are associated with poor prognosis [5].

The differentiation of primary and metastatic tumours of breast can be a diagnostic challenge. High suspicion of the metastatic tumour to the breast in a patient presenting with breast lumps with underlying malignancy necessitates further investigation as the clinical examination may not be able to differentiate primary breast lesions from metastases. To the best of our knowledge, there has never been a published case in the literature of primary mediastinal NETs metastasizing to the breast, hence we report this case as follows and outline the diagnostic workup for our patient. This work has been reported in line with the SCARE criteria [6].

## 2 Case report

**Fig. 1:**
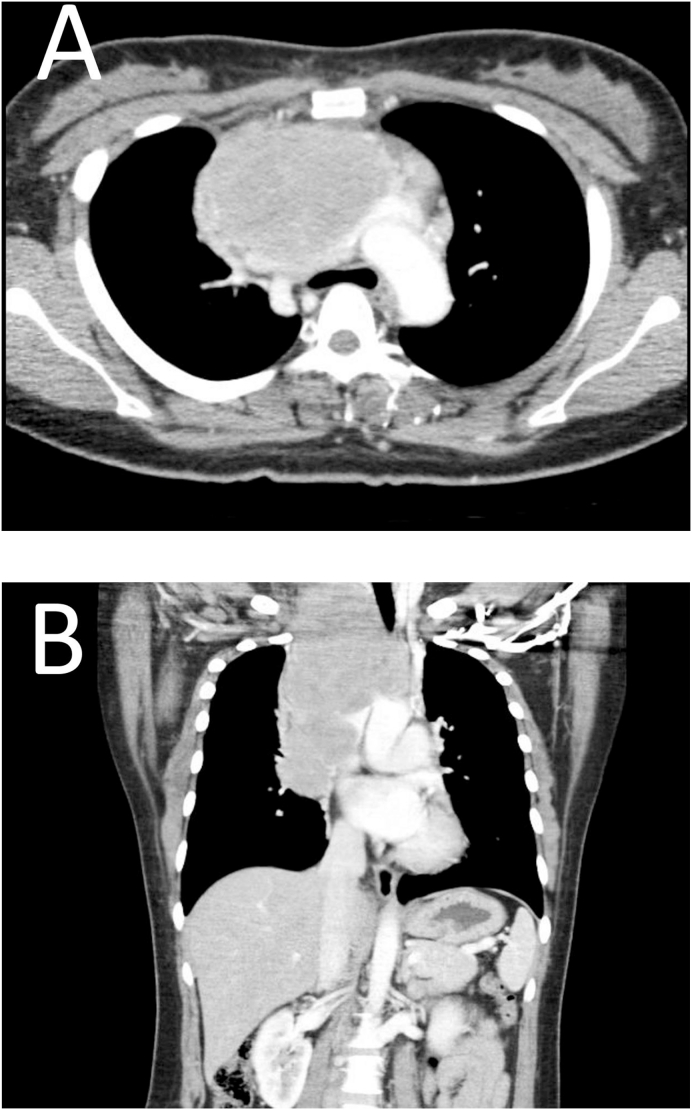
Chest CT showing a large mass at the anterior mediastinum in axial (A) and coronal (B) view.

A 26-year-old female presented to the Breast & Endocrine Clinic after being referred from a district hospital with an increasing anterior neck swelling over a period of 6 months with symptoms of superior vena cava syndrome. She was previously well with no known medical, surgical or genetic problem. On physical examination, the patient had a congested facial appearance, with a large hard fixed anterior neck mass. She was ambulating well with neither features of respiratory compromise nor signs and symptoms of carcinoid syndrome. Initial differentials include a large multinodular goiter or thyroid malignancy. Chest radiography showed a widened mediastinum. In view of the large nature of the mass, we opted for a computed tomography (CT) of the neck and thorax instead of an ultrasound neck, as the CT scan would be able to delineate the anatomy of the mass in relation to its surrounding structures. The CT thorax showed a huge anterior mediastinal tumour with mass effect to the surrounding structures associated with multiple cervical and mediastinal lymphadenopathy (Fig. 1). The thyroid, however, is grossly normal on the CT scan. The differential diagnosis has subsequently shifted to primary mediastinal lymphoma. A core biopsy of the neck mass was taken under ultrasonography guidance and histopathological examination showed nests of tumour cells, scant cytoplasm and salt and pepper chromatin. The tumour cells were diffusely positive for synaptophysin, chromogranin and the Ki-67 proliferative index is 50%. These constellations of finding were suggestive of high-grade small cell NETs.

As her tumour was unresectable, she was referred to our oncology team for chemotherapy. She had first completed 6 cycles of Etoposide and Cisplatin. However, the response was poor and she subsequently underwent a further 3 cycles of Irinotecan and Carboplatin. Follow up CT showed stable disease initially, but had turned into a disease progression a few months later. She subsequently underwent radiotherapy to the neck and mediastinum with a total of 30 Gy in 10 fractions.

**Fig. 2:**
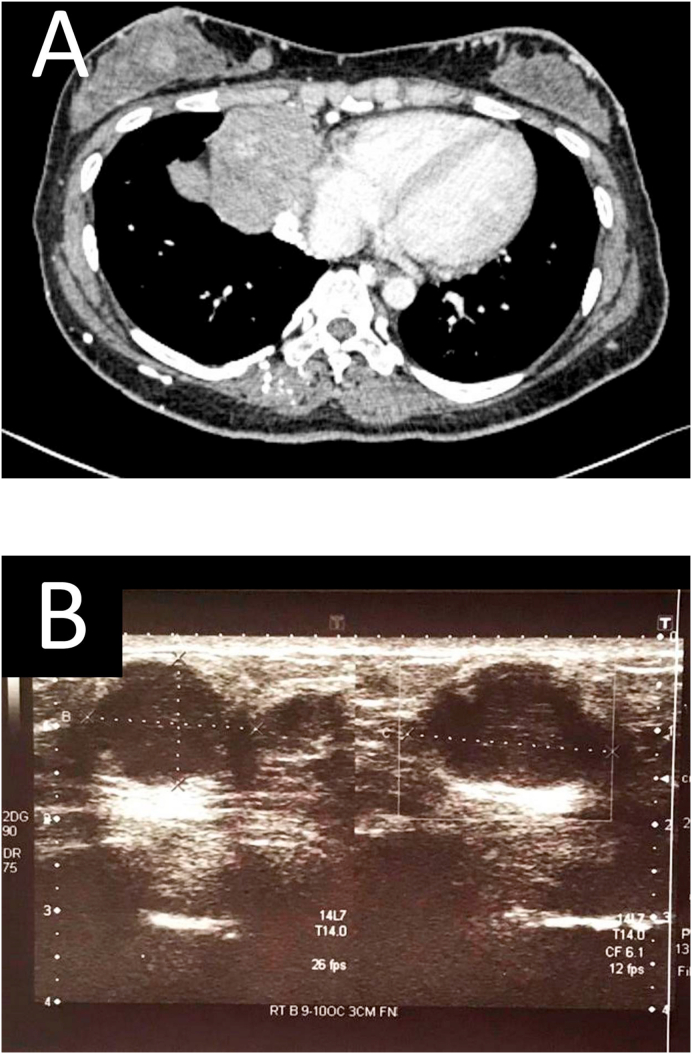
Chest CT revealing an enhancing right breast nodule (A). Ultrasound of breast (B) showing irregular hypoechoic breast lesions.

Restaging CT scan showed enlarging anterior mediastinal mass with multiple new enhancing bilateral breast lesions and hilar lymphadenopathy (Fig. 2A). Upon reviewing the CT scan with the patient in the clinic, she complained of enlarging bilateral painless breast lumps over the past 3 months which was not previously present. She denied family history of breast and ovarian malignancies. Clinical examination revealed multiple mobile breast lumps with a firm consistency over bilateral breast of varying sizes. There was no axillary lymphadenopathy. This clinical picture is typical of a breast fibroadenoma. However, with her underlying history of a mediastinal NET, the diagnosis of breast metastases was kept in mind. Sonographic assessment of the breast was done to further characterize the lesions and showed multiple oval-shaped hypoechoic lesions with irregular margins and posterior acoustic enhancement (Fig. 2B). These suspicious features may represent primary breast malignancy or breast metastases.

**Fig. 3:**
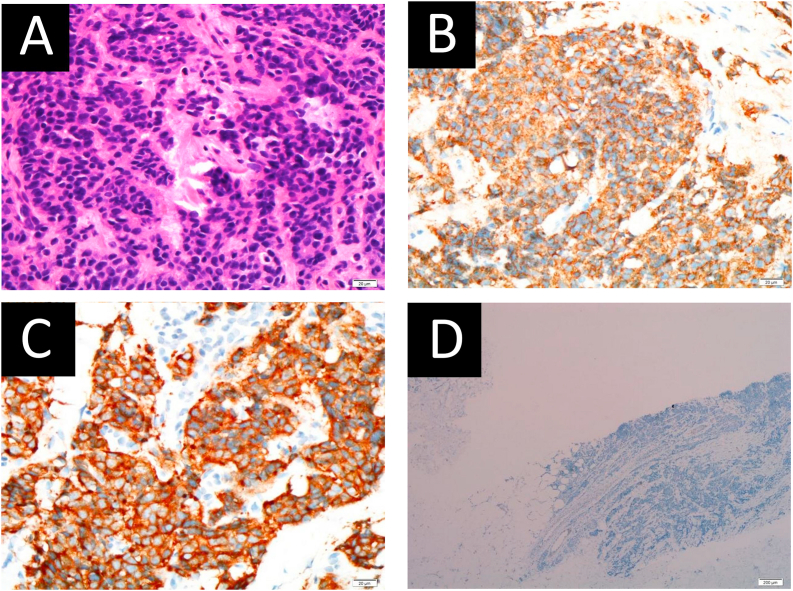
Microscopic examination shows a small blue cell tumor infiltration in sheets and nesting pattern with surrounding desmoplastic stromal reaction. The malignant cells (A) exhibit enlarged hyperchromatic nuclei and in-conspicuous nucleoli and scanty eosinophilic cytoplasm (haematoxylin-eosin staining x40). Immunohistochemical analysis was positive for (B) chromogranin A (magnification ×40), (C) synaptophysin (magnification ×40) but negative for (D) mammoglobin (magnification ×4). (For interpretation of the references to colour in this figure legend, the reader is referred to the Web version of this article.)

With the possible diagnosis of breast malignancy, core biopsy of the bilateral breast lesions was performed instead of fine needle aspiration cytology (FNAC). Histopathological examination of the lesions showed malignant cells infiltration in sheets and nesting patterns (Fig. 3A). Immunohistochemistry studies show that the malignant cells were positive for synaptophysin (Fig. 3B) and chromogranin (Fig. 3C), and the Ki-67 proliferative index was 50%. These cells were negative for mammaglobin (Fig. 3D). These features confirmed the diagnosis of metastatic NETs to the breast.

Despite the prompt initiation of second-line chemotherapy and radiotherapy, her tumour showed no signs of regression. She subsequently refused further treatment and was followed-up 3 monthly at our clinic to review her general well-being. She succumbed one year later due to neoplastic progression.

## 3 Discussion

Any breast pathology either cancerous or non-cancerous lesion requires a triple assessment to diagnose [7]. Besides history and clinical examination, standard radiological imaging techniques such as ultrasonography and mammography are useful to delineate these lesions. The commonest ultrasonography features of breast metastases are round or oval-shaped microlobulated and hypoechoic lesions [5]. This description, however, is very much similar to other suspicious primary breast lesions and does little to aid in the differentiation of the two. However, metastatic lesions tend to have posterior acoustic enhancement rather than shadowing, such as was in the case of our patient.

Digital mammography is another radiological assessment for breast lesions. However, it may be difficult to differentiate primary breast carcinoma from breast metastases. Metastatic lesions usually present as multiple, high density, round and circumscribed masses. They do not exhibit spiculated margins, skin or nipple retraction owing to the lack of desmoplastic reaction which occurs in invasive ductal carcinoma. Nonetheless, the diagnostic performances of it are variable and depend on certain factors such as dense breast tissue, which could potentially mask breast lesions [8]. Mammography was not performed in our patient due to the high density of breast in the younger age group.

Contrast-enhanced spectral mammography (CESM), a relatively new imaging technique based on the administration of an iodinated contrast medium and dual-energy exposure is of valuable role in differentiating primary breast cancer from breast metastases than standard digital mammography. This technique produces a low-energy image, superimposable to 2-dimensional digital mammography, and a recombined image obtained through subtraction with a high-energy image, allowing to emphasize breast areas with greater angiogenesis. CESM has a higher sensitivity than standard mammography in the detection of breast lesions [9].

Differentiating between a primary breast malignancy and metastases can be histologically challenging. Rodriguez-Gil et al. studied 7 patients with different primary tumours metastasizing to the breast and found that FNAC may provide a definitive diagnosis in the presence of a previously diagnosed extramammary malignancy [10]. However, this study did not include patients with breast metastases from NETs. Core needle biopsy is still preferred over FNAC for the diagnosis of breast lesions as it provides a more reliable diagnosis with the addition of ancillary immunohistochemical and molecular tests [11].

Histologically, the presence of intraductal or in-situ proliferation is usually strongly indicative of a primary breast lesion. It has been reported that primary breast NETs exhibit positive oestrogen and progesterone receptors and human epidermal growth factor-2 receptors would often be negative, giving a similar picture to luminal A breast cancer. NETs’ specific stains such as synaptophysin, chromogranin A and the Ki-67 proliferative index help to confirm primary breast NETs from other primary invasive breast carcinoma subtypes [12]. Mammaglobin staining could be useful in differentiating primary breast malignancy from other non-breast secondary metastases [13].

National Comprehensive Cancer Network recommends surgical resection for localized disease, resection followed by chemotherapy and/or radiotherapy for the locoregional disease [14]. However, treatment should be tailored according to individuals. The prognosis of high-grade mediastinal NETs is poor with a 5-year survival rate of 0% and median survival is only 13 months [2].

## 4 Conclusion

Metastatic NETs to the breast are extremely rare. We report the first case of metastatic primary mediastinal NETs to the breast. High suspicion of an index is needed for diagnosis when patients with known primary NETs present with suspicious breast lesions. Histopathological and immunohistochemistry staining are the mainstay of diagnosis.

## Ethical approval

Not related as this is a Case report.

## Source of funding

Not available.

### Authors’ contributions

KHC - literature search, manuscript draft and conceptual design of the work.

CHL - literature search, involvement in the management of the patient.

SZS - involvement in the management of the patient.

FH - contributed the conceptual design of the work.

SS - preparation of the histopathological figure and input.

## Registration of research studies

This is a Case report. No human participants were involved.

## Guarantor

Firdaus Hayati.

## Consent

Consent was obtained from the patient's next of kin.

## Provenance and peer review

Not commissioned, externally peer-reviewed.

## Declaration of competing interest

The authors declare that no relevant or material financial interests exist.

## Abbreviation

CT: Computed tomography
FNAC: Fine-needle aspiration cytology
NETs: Neuroendocrine tumours
CESM: Contrast-enhanced spectral mammography
